# The circadian clock in enamel development

**DOI:** 10.1038/s41368-024-00317-9

**Published:** 2024-09-06

**Authors:** Ke Wu, Xiaochan Li, Yunyang Bai, Boon Chin Heng, Xuehui Zhang, Xuliang Deng

**Affiliations:** 1 Department of Geriatric Dentistry, Peking University School and Hospital of Stomatology, Beijing, China; 2 4th Division, Peking University School and Hospital of Stomatology, Beijing, China; 3 Department of Dental Materials & Dental Medical Devices Testing Center, Peking University School and Hospital of Stomatology, Beijing, China; 4 National Engineering Research Center of Oral Biomaterials and Digital Medical Devices, NMPA Key Laboratory for Dental Materials, Beijing Laboratory of Biomedical Materials & Beijing Key Laboratory of Digital Stomatology, Peking University School and Hospital of Stomatology, Beijing, China; 5 Oral Translational Medicine Research Center Joint Training base for Shanxi Provincial Key Laboratory in Oral and Maxillofacial Repair Reconstruction and Regeneration The First People’s Hospital of Jinzhong, Jinzhong, China

**Keywords:** Pathogenesis, Molecular medicine

## Abstract

Circadian rhythms are self-sustaining oscillations within biological systems that play key roles in a diverse multitude of physiological processes. The circadian clock mechanisms in brain and peripheral tissues can oscillate independently or be synchronized/disrupted by external stimuli. Dental enamel is a type of mineralized tissue that forms the exterior surface of the tooth crown. Incremental Retzius lines are readily observable microstructures of mature tooth enamel that indicate the regulation of amelogenesis by circadian rhythms. Teeth enamel is formed by enamel-forming cells known as ameloblasts, which are regulated and orchestrated by the circadian clock during amelogenesis. This review will first examine the key roles of the circadian clock in regulating ameloblasts and amelogenesis. Several physiological processes are involved, including gene expression, cell morphology, metabolic changes, matrix deposition, ion transportation, and mineralization. Next, the potential detrimental effects of circadian rhythm disruption on enamel formation are discussed. Circadian rhythm disruption can directly lead to Enamel Hypoplasia, which might also be a potential causative mechanism of amelogenesis imperfecta. Finally, future research trajectory in this field is extrapolated. It is hoped that this review will inspire more intensive research efforts and provide relevant cues in formulating novel therapeutic strategies for preventing tooth enamel developmental abnormalities.

## Introduction

Key roles of the circadian clock in diverse biological processes Circadian rhythms, also known as the circadian clock, are highly-conserved and self-sustaining timing mechanisms within living organisms, which oscillate diurnally over 24 h. It is implicated in the regulation of a diverse array of physiological processes including hormone secretion, heart-beat, sleep-wake cycle, and body temperature.^1^ Such a time-based regulatory mechanism might have evolved in response to the diurnal cycle of light and darkness arising from the earth’s 24 h rotation. Light is correlated with energy harvesting together with DNA damage, while darkness is widely regarded as a phase for recuperation, energy storage and DNA repair.^2^ The evolution of the circadian clock also allows the temporal separation of incompatible processes in organisms to prevent antagonistic chemical reactions from occurring simultaneously, a typical example being redox oscillation.^3^

The clock mechanism located within the hypothalamic suprachiasmatic nucleus (SCN) of the mammalian brain is regarded as the “master clock”,^4^ while “peripheral clocks” are widely distributed in peripheral tissues and organs throughout the body.^5,6^ The master clock within the SCN acts as a pacemaker to synchronize the peripheral clock within peripheral tissues.^7^ In some conditions, such as changes in metabolism caused by temporal feeding restriction, peripheral clocks could be uncoupled from the central pacemaker in the SCN.^8^ It is also well-documented that the peripheral clock can oscillate independently without regulation by the SCN.^9,10^ In other words, the peripheral clock is just one oscillatory mechanism within cells at the transcriptional level,^11^ as there are other oscillatory mechanisms present such as oxidative phosphorylation (OXPHOS) and the cell cycle.^3^

The peripheral clock can be periodically synchronized to geophysical time mainly via the photoperiod of sunrise and sunset.^12^ Other known inputs to the peripheral clock include feeding and exercise,^13–15^ with studies having demonstrated that physical activity can modulate the rhythm of the clock machinery in skeletal muscles.^14,16,17^ Nutritional changes can also exert an effect on peripheral clocks via metabolic changes.^18^ Nevertheless, the biological clock can still oscillate even after stimuli (like the light-dark stimulus) is removed.^19^ The peripheral clock is known to be more easily disrupted by environmental stimuli than the master clock.^20^

Among the various stimuli, light is the most dominant stimulus to the endogenous clock that is input via the retina-hypothalamus tract.^9^ The photic information is received by photoreceptor cells in the retina, and then converted to electrical signals, which are transmitted by axons of the retino-hypothalamic tract, and finally transduced into a chemical signal that alters the SCN clock gene expression profile.^6^ The SCN rhythmic information can be transmitted to other brain regions and peripheral tissues via various types of outputs including neuronal connections, endocrine signals, and body temperature rhythms. Melatonin is a neuroendocrine hormone that is primarily generated and emitted by the pineal gland and serves as a photoperiod messenger.^21^ The target tissue responses to melatonin are mediated by melatonin receptors (MT1 and MT2). These are distributed in the brain as well as many peripheral tissues including the retina, lung, liver, and skin.^22–24^ Studies have shown that melatonin receptors are expressed in human tooth germs, which might indicate the presence of circadian rhythms in dental tissues.^25^ Nevertheless, melatonin is not the only chemical signal output pathway, as the central clock pacemaker may also exploit multiple signaling cues to synchronize the peripheral clock. These include cAMP, protein kinase C, glucocorticoids, Ca^2+^,^26^ corticotropin-releasing hormone (CRH), adrenocorticotropic hormone (ACTH),^27^ thyroid hormones and melatonin, all of which exhibit oscillations in serum levels based on a diurnal rhythm.^28,29^

The key circadian clock genes (CCGs) are closely intertwined with metabolism, immune function, cell proliferation, and signal transduction.^30^ Hence, disruptions in circadian rhythms or mutations in circadian clock genes are linked to the onset and progression of a diverse variety of diseases, including metabolic disorders,^31^ liver diseases, heart diseases,^32^ kidney diseases^33^ and cancer.^34^ Although some studies have shown that the circadian clock influences tooth enamel mineralization, the regulatory mechanisms still need to be elucidated.^19^

Circadian rhythms can be modulated by various therapeutic strategies including re-synchronizing the clock, modulating the output, or fine-tuning the central SCN clock.^35^ Recent research has demonstrated that circadian rhythms in the expression of anti-tumor immune components can be therapeutically exploited,^36^ indicating that regulation of the circadian clock may be a viable therapeutic strategy, particularly for diseases associated with disruption of circadian rhythms.^35^ This might be a hint of potential clinical applications of similar therapeutic strategies in other disease models. However, it is still unclear to what extent such therapeutic strategies could ameliorate the effects of disrupted circadian rhythms.^37^

## Tooth enamel

### The structural and functional properties of tooth enamel provides evidence for the key role of the circadian clock

Tooth enamel has a distinct hierarchical microstructure known as enamel rods that are made up of hundreds of lath-like crystallites.^38^ Interspersed between the enamel rod crystallites is the amorphous phase. There are two intergranular phases: magnesium amorphous calcium phosphate and mixed-phase iron oxide.^39^ The amorphous phase of enamel controls the demineralization rate and renders it more resistant to acid attack. Hence, the existence of these two distinct phases in adult tooth enamel significantly contributes to its remarkable physical, mechanical and chemical properties, and is strongly suggestive that these may develop at different cyclic phases (i.e. day/night) of the circadian clock.

**Fig. 1:**
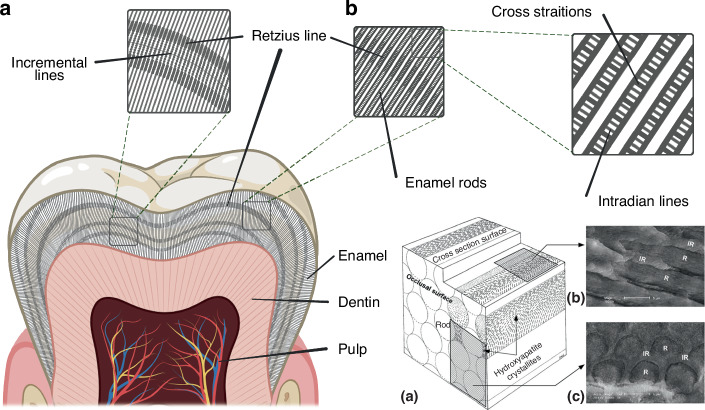
Microstructures of mature tooth enamel. **a** Incremental lines in tooth enamel, including cross striations, Retzius lines and intradian lines; **b** Schematic diagram of enamel rods (a) and electron microscopy images of enamel at different orientations: parallel to the rods (b) and perpendicular to the rods (c) (Adapted from Schneider et al. 2008)

Other observed microstructures of mature tooth enamel are classified as incremental features, including the cross-striations, the intradian lines, the Retzius lines, the stress lines and the laminations^40^ (Fig. 1), which are different classes of incremental lines, with some of these being more clearly observable after fluorochrome injections.^41–43^ These structural features are closely related to the formation processes of teeth enamel and can therefore be regarded as its cyclical growth patterns,^42^ which is strongly suggestive of the role of the circadian clock in its development. The cross-striations are incremental lines with light and dark bands that cross the enamel prisms perpendicularly with a spacing interval of about 3–6 µm. These represent the daily secretory phases of enamel-forming cells, known as ameloblasts.^42^ The intradian lines are fine bands divided by cross-striations into two or three segments, representing a shorter enamel secretion period of less than 24 h.^40^ However, the exact rhythmicity of the intradian lines remains obscure.^43^ The Retzius lines are internal long-period features found in primate enamel, and the number of cross-striations between two successive Retzius lines indicates the interval of Retzius line formation.^44^ The spacing interval of the Retzius lines has been reported to be between 4 and 150 μm and is observed to decrease from the center to the periphery.^45^ The laminations are spaced similarly to the cross-striations and appear parallel to the Retzius lines, and these may also be found in aprismatic enamel as well. However the relationship between laminations and cross-striations remains unclear.^40,43,46^

Apart from perturbations of circadian rhythms, any insult or disruption that occurs during enamel formation may also leave growth marks on mature enamel that are manifested as wider or darker bands, referred to as stress lines.^47^ The neonatal line is one of the most prominent stress lines, which is evidence of sudden changes in the environment during the birth process.

### Cyclical pattern of ameloblast function and metabolism in tooth enamel formation provides evidence for the key role of the circadian clock

Ameloblasts, or enamel-forming cells, are the key players in enamel formation and mineralization. The development and mineralization of enamel is a complex and precisely-orchestrated process that is tightly regulated by ameloblasts.^48^ Amelogenesis is initiated when the ameloblasts secrete enamel matrix proteins (EMPs), forming a hydrated protein-rich matrix. Subsequently, this matrix is then proteolytically degraded and removed by ameloblasts, to facilitate enamel mineralization.^49^ However, compared to bone which is a mineralized tissue with self-repair functions, mature enamel is acellular and hardly undergoes self-repair after damage.^50^

**Fig. 2:**
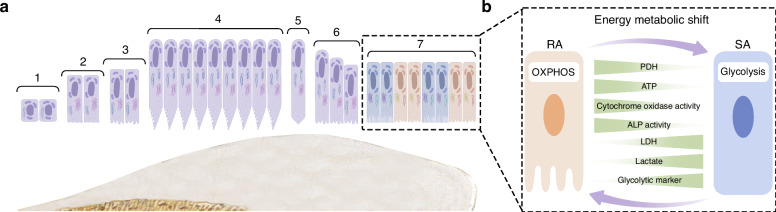
The life cycle of ameloblasts. **a** The life cycle of ameloblasts, includes the pre-maturation stage (1–3), secretory stage (4), transition stage (5-6), maturation stage (7) and post-maturation stage. Among them, maturation stage contains ruffled end ameloblast (RAs) and smooth end ameloblasts (SAs); **b** The energy metabolism in RA/SA transition

The life cycle of ameloblasts includes the pre-maturation stage, secretory stage, transition stage, maturation stage and post-maturation stage (Fig. 2a). The secretory and maturation stages, in particular, are primarily responsible for enamel formation. The EMPs are secreted by the secretory stage ameloblasts (S-ABs), which play a crucial role in enamel development by facilitating its calcification. After undergoing the transition stage, the S-ABs differentiate into maturing ameloblasts (M-ABs), which help transport minerals, regulate enamel mineralization and modulate protein degradation and absorption.^50–52^

During the maturation stage, ameloblasts cyclically alternate between two morphological phenotypes, which are known as ruffle-ended ameloblasts (RAs) and smooth-ended ameloblasts (SAs) respectively (Fig. 2b). The alternation between these two morphological phenotypes occurs 5 ~ 7 times.^53–55^ In the case of rodent ameloblasts, the RA phase lasts for about 8.5 h, while the SA phase lasts for about 2 h.^56^

It was observed that maturation-stage ameloblasts exhibited distinctively different subcellular localization and morphology of mitochondria, as compared to secretory-stage ameloblasts. During the secretory stage, mitochondria are mainly located between the cell nucleus and the proximal or basal pole.^57^ Their shape is predominantly elongated and is variable in length.^57^ As for the mitochondria of maturation stage ameloblasts, these are primarily located at the proximal pole within the infranuclear region or adjacent to the distal ruffled border, covering about half the way from the apical cytoplasm to the nucleus and continuing to the Golgi region,^54^ which may contribute to ion movement.^58^ The morphology of the mitochondria during the maturation-stage is mainly spheroidal-shaped, with some internal “helices” described as DNA structures.^57^

Some studies have indicated that as the ameloblasts transition from the secretory to the maturation stage, the basal mitochondria increase in size and number, as well as tend to show inclusions such as helical structures.^59,60^ A study based on HAT7 cells reported that RAs preferentially utilize OXPHOS for energy supply, while SAs rely on glycolysis-dominant energy metabolism.^61^ This could be because RAs require more ion transport, endocytosis and other functions, so their energy demand is higher than that of SAs. Some reports have suggested that the energy metabolic state is an important determinant of the RA/SA phenotype,^61^ as RA would undergo a transition to SA once its metabolism is exhausted.^62^ However, the regulatory mechanisms of the phenotype switch by maturation-stage ameloblasts need to be further elucidated.^63,64^

### Enamel matrix proteins

**Fig. 3:**
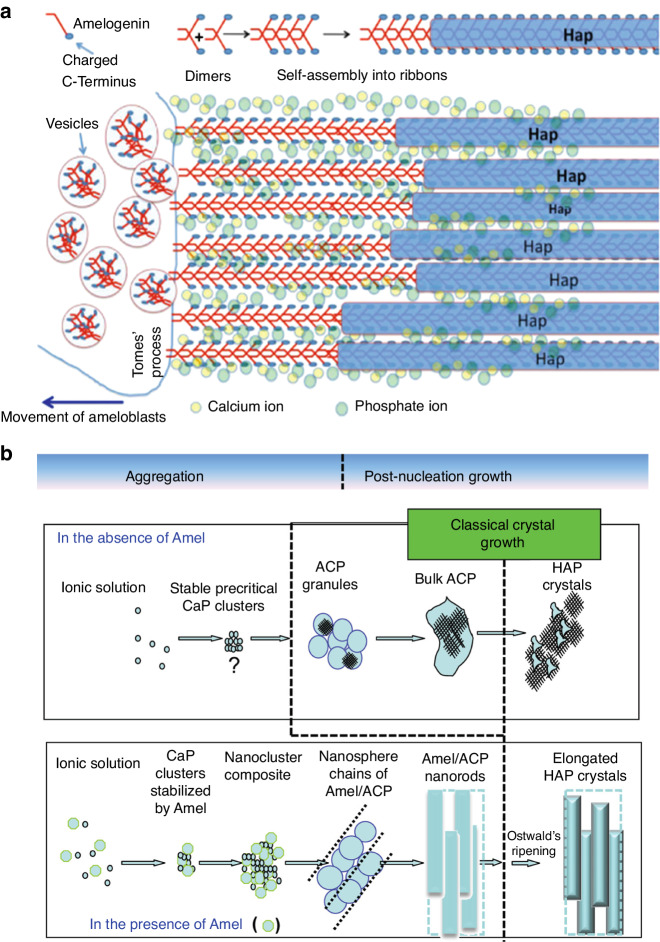
Aligned AMELX nanoribbons act as a template for guided apatite crystal growth and serve as a precursor to enamel rods. **a** Enamel matrix protein deposition facilitates mineralization and calcification, with aligned Amelx nanoribbons acting as a template for guided apatite crystal growth and serving as a precursor to enamel rods. Ca^2+^ and PO_4_^3−^ stabilize the nanoribbon structure by forming ion bridges between AMELX dimers (Adapted from Lacruz et al.,^48^); **b** Amelx is essential for the formation of a hierarchically HAP crystal microstructure (Adapted with permission from Yang et al., 2010, copyright 2010 American Chemical Society.)

Mature enamel contains very few organic materials such as proteins and peptides (less than 1%), whereas proteins make up the bulk of the developing enamel matrix. During the secretory stage, ameloblasts synthesize and secrete structural enamel matrix proteins (EMPs), including amelogenin (AMELX), ameloblastin (AMBN), enamelin (ENAM)^60,65,66^ and various proteinases.^67^ Among these up to 90% is AMELX,^68^ which forms AMELX nanospheres through the utilization of its self-assembly properties. Aligned AMELX nanoribbons could act as a template for guided apatite crystal growth and serve as a precursor to enamel rods^69^ (Fig. 3). Besides AMELX, other proteins also play crucial roles in amelogenesis. For example, AMBN is a key cell adhesion molecule during ameloblast development and differentiation,^70^ while ENAM is essential for apatite nucleation.^71^

Various proteinases are also involved in amelogenesis. Enamelysin (Matrix metallopeptidase 20, MMP20) and kallikrein-related peptidase 4 (KLK4) are two key extracellular proteinases expressed during amelogenesis that cleave structural proteins and function in enamel maturation and mineralization.^65^ MMP20 is expressed mainly during the secretory stage,^65,72,73^ while KLK4 is predominantly expressed during the maturation stage.^72,74,75^ These two proteinases degrade and remove EMPs preferentially and selectively from HAP in vitro.^76^ The secretory profiles of most of these EMPs and proteinases follow a cyclical pattern,^19,77,78^ which strongly indicate that the circadian clock is implicated in amelogenesis by modulating the secretion of these proteins by ameloblasts.

### Mineralization and ion transport during tooth enamel development

Maturation-stage ameloblasts facilitate ion transport towards the enamel layer.^51^ Passive and active pathways of ion transport are both present. The former is referred to as the passive paracellular/intercellular movement through “leaky” junctions^51,76^ or the passive transcellular pathway; while the active transport pathways involve ion transporters, channels and pumps.^66,79,80^ Ions from the circulation, such as Ca^2+^ and HCO_3_^-^, diffuse across the papillary layer and are actively transported into polarized ameloblasts via ion exchangers and pumps located at their basolateral membrane,^81,82^ which are implicated in bicarbonate production (*Car2*), bicarbonate transport (*Slc4a4*) and enamel matrix endocytosis (*Lamp1*).^66^ These various ion exchangers are known to be differently expressed during the light/dark cycle,^78^ thus implicating the circadian clock also regulates ion transportation. Ca^2+^ is mainly transported actively through the store-operated Ca^2+^ entry (SOCE) channels. SOCE entails Ca^2+^ release from intracellular pools followed by Ca^2+^ entry. The Ca^2+^ release-activated Ca^2+^ (CRAC) channels,^83^ which are primarily composed of ORAI calcium release-activated calcium modulator 1 (ORAI1), stromal interaction molecule 1 and 2 (STIM1 and STIM2), are the best-characterized SOCE channels and they are significantly upregulated in maturation stage ameloblasts. Intracellular [Ca^2+^] signaling plays an important role in both enamel mineralization and peripheral clock timekeeping,^84^ therefore it could be an essential intermediary between the circadian clock and amelogenesis.

To date, much progress has already been made in the understanding of amelogenesis as well as the functional properties of mature enamel so far. However, partly due to enamel’s highly hierarchical structure and the complexity of its chemical composition which still needs to be further explored, there has been limited success in repairing enamel lesions or synthesizing enamel in vitro.^38,39^

## Role of the circadian clock in enamel development

### The molecular mechanisms of circadian clocks

**Fig. 4:**
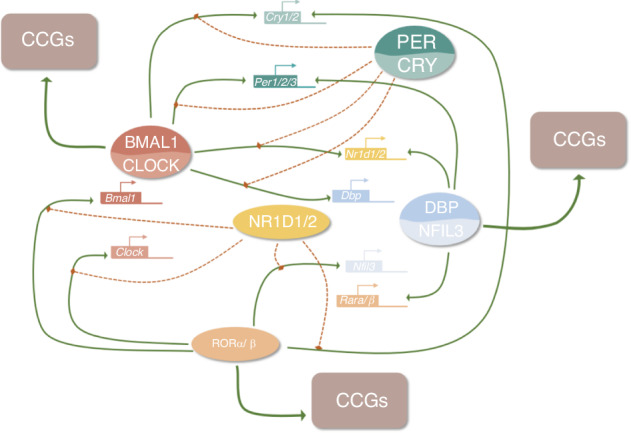
The molecular mechanism of TTFLs

The autoregulatory cell-autonomous transcriptional-translational feedback loops (TTFLs) in mammals generate the molecular mechanisms of cellular circadian clocks.^30,85^ This cellular clock system is made up of several genes, including the core clock genes *circadian locomotor output cycles kaput* (*CLOCK)* and *brain and muscle ARNT-like 1* (*BMAL1)* which handle positive regulation, as well as *period (PER1*, *PER2)* and *cryptochrome* (*CRY1*, and *CRY2)* which handle negative regulation.^30,85,86^ The general molecular mechanisms of TTFLs are illustrated in Fig. 4. CLOCK together with BMAL1 heterodimerize to form the CLOCK: BMAL1 heterodimers that bind directly to the regulatory elements (E-boxes) of a set of rhythmic genes encoding the repressor proteins period (Per1, Per2, Per3) and cryptochrome (Cry1, Cry2) to promote transcription.^86,87^ Other clock proteins regulate the expression of Clock and Bmal1,^88^ which is linked to three transcriptional autoregulatory feedback loops. The first feedback loop is made up of PER and CRY, and in mammals, PER heterodimerizes with CRY, inhibiting CLOCK: BMAL1 transcriptional activity and repressing their gene transcription.^87,89–91^ The second feedback loop is composed of REV-ERB (encoded by the genes *Nr1d1/Nr1d2* and *Rora/b*) and retinoic acid-related orphan receptor (ROR) proteins.^92,93^ REV-ERB and ROR have opposite effects as they bind to retinoic acid response elements (RREs) on gene promoters, with REV-ERB inhibiting gene transcription, while ROR promotes gene transcription.^94–96^ The target genes of REV-ERBs/RORs include *BMAL1, CLOCK*,^96^ *and CRY1*,^97^ exerting both negative and positive effects on the expression of core clock genes and other clock-controlled genes (CCGs), especially metabolism-related genes.^98,99^ REV-ERB gene expression is in turn regulated by CLOCK, BMAL1,^99^ and PER2,^100^ thus forming a feedback loop. Another feedback loop is related to D-site-binding protein (DBP)^101^ and nuclear factor interleukin-3 regulated (NFIL3),^102^ which dimerize and bind to D-box elements on target genes, including *Per1/Per2*,^103^ *Nr1d1/Nr1d2*, and *Rora/Rorb*. The timing of the PER/CRY negative feedback loop is also influenced by the DBP/NFIL3 loop.^104^ Among these three loops, the REV-ERBs/ROR loop acts as a key output mediator, playing a key role in regulating genes related to metabolism,^98^ while DBP/NFIL3 influences the circadian transcription of many other target genes and responds to cellular signals.^105,106^

The binding of the CLOCK: BMAL1 complexes is also affected by other signaling molecules. For example, adenosylhomocysteinase (AHCY) is required for BMAL1 genome-wide recruitment to chromatin and subsequent circadian transcription,^106^ which implies that the circadian clock system might have regulatory crosstalk with other physiological systems such as energy metabolism.^106^ Additionally, TTFLs can be directly affected by extracellular stimuli via intracellular transduction by second messengers such as calcium and cAMP.^107,108^ Calcium has been shown to act on TTFLs through the calcium/cAMP-dependent transcription factor (CREB).^84^ Therefore, these three feedback loops have many complex interactions with the core clock genes and CCGs, as well as crosstalk with other systems that are essential for cellular activities.

*BMAL1, CLOCK, PER1* and *PER2* expression can be detected in dental tissues during tooth development and display regular oscillatory expression patterns in tooth germs.^19,78,109^ Circadian clock gene expression could be influenced by photoperiod stimulus,^110^ with the expression levels of *Bmal1* and *Clock* peaking during the light phase.^77,110^ In an in vitro study, periodic opposite oscillations of *Per2* and *Bmal1* within 48 h can be detected in ALC cells after synchronization,^111^ with *BMAL1* and *PER2* being reciprocally expressed in ameloblasts and transferred from the nucleus to cytoplasm every 12 h.^19^

### Circadian clock regulation throughout the lifespan of ameloblasts

Existing studies provide multiple lines of evidence that circadian rhythms influence ameloblast behaviour, including cell differentiation, cell morphological and functional transitions, energy metabolism, pH regulation and intercellular connections.

The differentiation of ameloblasts is known to be perturbed by circadian rhythm disruption. Studies have shown that abnormal circadian rhythms in pregnant mice can lead to delayed tooth germ development in their offspring. Histological observations have shown that upon circadian clock disruption, ameloblasts could not form a normal tall columnar structure but retained a short columnar structure, with the polarity of the corresponding odontoblasts being delayed as well.^112^

Evidence suggests that the maturation phase of amelogenesis has a rhythmic pattern, and this pattern may be related to circadian rhythms, as implied by its incremental features. AMELX is regulated by the core clock genes (*Bmal1, Clock, Per1*, and *Per2*), which oscillate at both the RNA and protein levels in a 24 h cycle.^109^ *Amelx* expression in 2-day postnatal mouse molars was measured every 4 h throughout a total duration of 48 h, with oscillations lasting approximately 24 h. The acrophase of AMELX level occurs during the light period, while the bathyphase occurs during the dark period,^78^ which exhibits reciprocal oscillations comparable to the expression of clock genes. Besides *Amelx*, *Ambn* and *Amtn* have also been detected to be rhythmically expressed during enamel development.^77^ During the dark period, however, the expression of maturation-stage specific genes involved in bicarbonate production (*Car2*), bicarbonate transport (*Slc4a4*) and enamel matrix endocytosis (*Lamp1*) are upregulated.^78^

The transcription factors runt-related transcription factor 2 (RUNX2) and distal-less homeobox 3 (DLX3), regulate the rhythmic expression of several ameloblast-specific genes throughout a diurnal period of 24 h, via interaction with the core clock genes.^113^ Being regulated by the circadian rhythm, periodic changes in RUNX2 expression in ameloblasts play a key role in inhibiting secretory phase gene expression (e.g. AMELX and ENAM) while upregulating expression of maturation stage genes, such as KLK4.^113^ In contrast, DLX3 is upregulated during the secretory stage, which is critical in promoting the expression of AMELX and ENAM, while being downregulated during the maturation stage.^113^

Synchronizing signals from the central clock may also affect amelogenesis. Melatonin likely regulates enamel development because the melatonin 1a receptor (Mel1aR) is expressed by secretory ameloblasts as well as other enamel-forming cells such as the cells of the stratum intermedium and stellate reticulum.^25^ As previously mentioned, SAs are primarily fueled by glycolysis while RAs are primarily fueled by OXPHOS for their energy metabolism. It has been proposed that the redox system exhibits an autonomous oscillation that interacts with the rhythmic oscillation of gene transcription within cells.^3^ This mechanism likely exists in ameloblasts, but more research is required to confirm this.

*Clock* also regulates cell adhesion in ameloblasts. *Per2* knockdown was reported to alter the subcellular localization of key markers of cell adhesion like E-cadherin in ameloblast-lineage cell (ALC) line, which would imply that *Per2* knockdown affected ameloblast organization and secretion.^111^

**Fig. 5:**
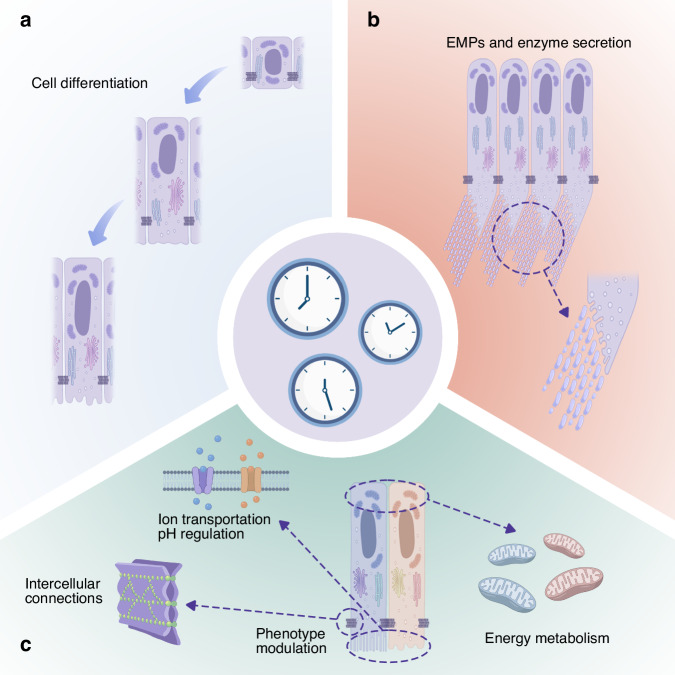
Possible mechanisms by which circadian clocks regulate ameloblasts and facilitate amelogenesis: **a** The differentiation of ameloblasts; **b** Enamel matrix protein and enzyme secretion; **c** Ion transportation, cell adhesion, energy metabolism and regulation of morphology

Cell differentiation, cell morphological and functional transitions, energy metabolism, pH regulation and intercellular connections are all rhythmically regulated during enamel development. Effective coordination between these oscillators may be required to ensure normal ameloblast function and the formation of well-developed tooth enamel. A summary of section 3.2 is presented in Fig. 5.

## The potential detrimental effects of Circadian rhythm disruption on enamel development

### Abnormal enamel development: enamel hypoplasia

Enamel hypoplasia (EH) is a quantitative dental enamel defect defined as a region of reduced enamel thickness with a smooth outline caused by disruptions in enamel formation during tooth development, which can be caused by inherited disorders or environmental insults.^114,115^ EH, caused by genetic factors is termed amelogenesis imperfecta (AI),^115^ and if the developmental defect is caused by systemic origins affecting one or more molars, it is termed as molar-incisor hypo-mineralization (MIH).^116,117^ Various oral healthcare problems are associated with EH such as poor oral hygiene, unsatisfactory aesthetic appearances, impaired masticatory function, dental sensitivity, increased dental care costs and requirement for lifelong extensive restorative care, in addition to detrimental effects on social interactions, which could severely impact the life quality of patients.^118,119^ A previous review summarized various EH risk factors, such as maternal and infant nutrition, premature birth, infantile convulsions and infection.^114^ EH attributable to inherited genetic disorders is quite rare, whereas EH caused by environmental or epigenetic factors is more common. It has also been suggested that compared to genetic factors, environmental risk factors may also exert a greater influence on the occurrence of EH in primary maxillary incisor teeth.^120^

Animal experiments have confirmed that circadian rhythm disruption leads to enamel hypoplasia in vivo. Because tooth development occurs during both the embryonic and postnatal periods, disruptions in either the maternal or postnatal circadian rhythms may disrupt enamel development. Maternal circadian rhythm disruption may affect their offspring. Studies have shown that neonatal mice exposed to all-dark or all-light environments have delayed enamel development, indicating that circadian rhythm disruption caused by photoperiod perturbations can impact amelogenesis.^112^ This evidence thus validated that circadian rhythm disruption of either the postnatal or prenatal phase can exert negative effects on amelogenesis.

It has been validated that environmental circadian disruption leads to the dysregulation of circadian gene expression in vivo. Abnormal circadian rhythms have been reported to disrupt the formation of mandibular first molar germs in mice, characterized by reduced protein expression levels of BMAL1 and PER2, as well as decreased immunohistochemical staining of BMAL1 and PER2 in ameloblasts.^111^ Neonatal mice exposed to an all-dark or all-light environment also exhibited significant changes in their expression level of melatonin receptors (MTs). Some studies have shown that the injection of MT2 selective inhibitor 4P-PDOT into E16 maternal mice results in abnormal teeth mineralization in their newborns but has no significant effect on the morphology of the tooth germ.^112^ Electron microscopy has shown that there are abnormalities in multiple organelles, including the disappearance of mitochondrial cristae and vacuolar degeneration of cells, but the underlying mechanisms need to be elucidated. Additionally, the light-dark photoperiod also affects the expression of melatonin receptors. Neonatal mice exposed to full dark or full light display significant changes in melatonin receptor (MTs) expression.^112^

To date, several studies have revealed the mechanisms of circadian rhythm disruption associated with EH at the histological and molecular levels. Electron microscopy imaging revealed some obvious voids among ameloblasts and increased necrosis of ameloblasts.^112^ Molecular dysregulation in ameloblasts was also observed, which is referred to as a downregulated expression of EMPs (i.e. AMELX), which in turn leads to reduced enamel matrix deposition.^111^ Apart from EMPs, other proteins that are essential for enamel cell activities such as cytokeratin 14 (CK14) and filamentous actin (F-actin) were also observed to be downregulated.^111^

It has also been demonstrated that targeting clock genes affects amelogenesis. *Per2* knockdown inhibits ameloblast-lineage cell (ALC) differentiation and also affects ALC cell adhesion by altering the subcellular localization of E-cadherin.^111^ *Nr1d1* over-expression resulted in the up-regulation of *Amelx* and the down-regulation of *Mmp20* and *Klk4*.^113^ Meanwhile, overexpression of *Bmal1* can lead to up-regulation of RNA expression levels of *Amelx* and *Klk4*, while overexpression of *cry1* had the opposite effect.^113^

Animal studies have validated that *Mmp20* or *Klk4* mutation is associated with non-syndromic AI, which is manifested as teeth enamel being not fully mineralized.^121^ *Mmp20*-null mice exhibit this phenotype with hypoplastic enamel, and the hypoplastic enamel would delaminate from the dentin soon after tooth eruption.^122^ *Klk4*-null mice exhibited an enamel hypomaturation phenotype and the enamel would abrade or fracture above the DEJ soon after tooth eruption.^123^ Changes in the expression levels of *Klk4* are closely associated with DLX3 over-expression or knockdown.^124^ As mentioned previously, the transcription factor DLX3 is regulated by core clock genes, thus giving rise to the hypothesis that circadian rhythm disruption might lead to the dysregulation of enamel proteinases like KLK4 via changes in DLX3 expression.^113^

In vivo, photoperiod perturbations might also affect the peripheral circadian clock via SCN and its hormonal output pathway. Pregnant mice treated with melatonin receptor antagonists exhibit enamel tissue deficiency in their offspring that is associated with downregulated expression of MTs and AMELX.^112^ Nevertheless, incremental lines can still be observed in mice dentin, even though SCN is partly or completely damaged. This evidence thus indicates that the peripheral clock and tissue-specific circadian rhythm in teeth might be more independent than previously assumed.^125^ Hence, it is reasonable to postulate that the same condition might also exist in enamel development.

**Fig. 6:**
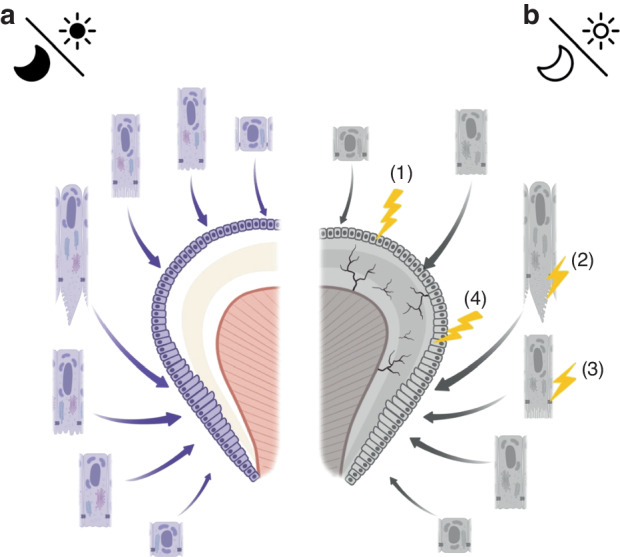
Possible mechanisms by which abnormal circadian rhythms negatively affect enamel formation: **a** Normal circadian rhythms facilitate normal amelogenesis, with the oscillations being well-coupled; **b** Dysregulation of circadian rhythms may cause delayed ameloblast differentiation (1), as well as downregulated expression of EMPs leading to reduced enamel matrix deposition (2), abnormal intercellular connections (3), ultimately resulting in delayed and defective enamel development (4)

As previously stated, circadian rhythms may integrate multiple independent oscillators during enamel development, a disruption or loss of rhythm may cause these oscillators to lose their synchronization. Although their respective functions may not be significantly affected, the coordination and synchrony with each other might go out of balance. As a result, antagonistic biological processes occur simultaneously, or the functional protein is not in the optimal environment to function properly, which may be the reason why enamel can still form but developmental abnormalities occur. Much further research is still required to rigorously characterize the potential negative effects of circadian rhythm disruption on enamel development. A summary of section 4.1 is presented in Fig. 6.

### Treatment of EH

Although a number of etiological hypotheses for EH have been postulated and the mechanisms have been revealed, the treatment for EH is still symptomatic, which means that the abnormal enamel can only be restored but not recovered. The treatment of enamel hypoplasia is a lifelong management, which requires the participation of multidisciplinary specialists in pediatrics, endodontics, periodontology, prosthodontics and orthodontics. This lifelong management requires a step-wise treatment plan, starting with the most conservative treatment regimen. The main treatment goals for EH patients are to prevent pain and early tooth loss, restore the appearance, function and aesthetics of dentition, protect tooth tissue and improve the quality of life.^126^

The main treatment modalities include oral hygiene instruction, dietary guidance, tooth desensitization, tooth bleaching and restorative treatment. Among these, the treatment modality that is closest to the etiological treatment might be applying Casein phosphopeptide–amorphous calcium phosphate (CPP–ACP) paste.^127,128^ Although this treatment could aid enamel remineralization, it still cannot exert any effect on new enamel formation. Rehabilitative treatment can improve the function and aesthetic appearance of the tooth, which includes the use of direct or indirect composite resin, veneer, crown, fixed partial denture, removable partial denture, and implant.^116^ The treatment of the severe symptoms is quite complicated, and due to the abnormal properties of enamel, the process of restoration treatment also faces many challenges. For example, EH patients’ teeth are brittle so that sufficient resistance and retention cannot be easily approached and it is difficult to obtain a good bonding effect. EH patients are more susceptible to caries, leading to more challenges in restoration or maintenance. Some patients have insufficient intermaxillary distance and clinical crown height due to severe wear, which requires crown lengthening surgery.^129^

Most evidence on AI treatment is from case reports and lacks randomized clinical trials, which means the quality of the evidence is unsatisfactory.^116,129^ Hence, treating EH by using an evidence-based approach is difficult. Long-term oral treatment also imposes financial and care burdens on EH patients and their families.

Therefore, we conducted this review to provide novel insights into the understanding of tooth enamel development. The role of circadian rhythms in enamel development could be a possible future research direction, which could offer new perspectives on novel treatment and preventive strategies for EH.

## Conclusion and future outlook

The scientific literature has validated the key roles of the circadian rhythm in a diverse multitude of physiological functions. Normal circadian rhythms are conducive to maintaining normal physiological processes, while circadian rhythm disruption is associated with many diseases. With regards to enamel development, the unique structural morphology of enamel with “incremental lines” indicates that enamel development is rhythmic in nature. While previous studies have revealed that circadian rhythm can regulate enamel formation and plays a key role in this process, there are still many molecular mechanisms to be elucidated.

Enamel hypoplasia leads to severe oral healthcare problems that affect the life quality of patients.^118,119^ Understanding the various signaling pathways and biological processes that are critical to enamel-forming cells might help avoid disruption of amelogenesis, and reduce the possibility of enamel hypoplasia occurring. This review thus outlines the key roles of circadian rhythms in amelogenesis, summarizing current available scientific evidence and extrapolating future research trajectory in the field. The expression of clock genes is examined in ameloblasts and tooth germs. Accumulated scientific evidence has indicated the critical roles of circadian rhythms in several physiological processes that are essential for various cellular functions, such as mitochondrial morphology changes, energy metabolism and cytoskeletal formation, all of which exert profound effects on ameloblasts that are primarily responsible for amelogenesis. Nevertheless, whether there is regulatory crosstalk between these systems and circadian rhythm in enamel development still needs to be further clarified.

More research is needed to determine whether all of the oscillations observed during tooth enamel development are circadian in nature, and whether or not there is the presence of independent oscillation mechanisms. The circadian clock is also a potential central regulator of these oscillators, and the specific integration mechanisms and coupling mode if these exist, would certainly merit further investigations. It is critical to understand the mechanisms by which normal circadian rhythm ensures normal enamel development, and whether stronger circadian rhythms could facilitate optimization of enamel formation.

In addition, most studies on tooth development and circadian rhythm are mostly based on mice or rodents, but not other mammals as animal models. However, tooth development in rodents is quite different from that of humans, and their biological rhythms also differ greatly from that of humans. The present study did not have the opportunity to critically examine other animal models with regards to biological rhythm and tooth development data because these are lacking within the scientific literature. Further research is thus needed to fill this gap.

To date, although several studies have already validated the circadian basis of amelogenesis, the roles of circadian rhythm in enamel development still need to be delineated further. It is hoped that this review will inspire more research efforts in this field, as well as provide relevant cues in establishing novel therapeutic strategies for clinical interventions to prevent developmental abnormalities of tooth enamel.
